# Key factors capturing the willingness to use automated vehicles for travel in China

**DOI:** 10.1371/journal.pone.0298348

**Published:** 2024-02-16

**Authors:** Yongjiang Zhou, Hanying Guo, Hongguo Shi, Siyi Jiang, Yang Liao

**Affiliations:** 1 School of Automobile and Transportation, Xihua University, Chengdu, Sichuan, China; 2 School of Transportation and Logistics, Southwest Jiaotong University, Chengdu, Sichuan, China; Shandong University, CHINA

## Abstract

With the continuous advancement of technology, automated vehicle technology is progressively maturing. It is crucial to comprehend the factors influencing individuals’ intention to utilize automated vehicles. This study examined user willingness to adopt automated vehicles. By incorporating age and educational background as random parameters, an ordered Probit model with random parameters was constructed to analyze the influential factors affecting respondents’ adoption of automated vehicles. We devised and conducted an online questionnaire survey, yielding 2105 valid questionnaires. The findings reveal significant positive correlations between positive social trust, perceived ease of use, perceived usefulness, low levels of perceived risk, and the acceptance of automated vehicles. Additionally, our study identifies extraversion and openness as strong mediators in shaping individuals’ intentions to use automated vehicles. Furthermore, prior experience with assisted driving negatively impacts people’s inclination toward embracing automated vehicles. Our research also provides insights for promoting the adoption of automated vehicles: favorable media coverage and a reasonable division of responsibilities can enhance individuals’ intentions to adopt this technology.

## 1 Introduction

With the full development of image recognition, artificial intelligence, and high-performance computers, the rules of the road will change with the spread of automated vehicles (AVs). To facilitate the research and development of automated vehicles, the Society of Automotive Engineers (SAE) established standards for the level of automation in January 2014. The standard classifies automated vehicles into six classes, L0 to L5, where L0 to L2 requires the driver to monitor the driving environment, while L3 to L5 are monitored by an Automated driving system [1]. In April 2021, SAE defined and differentiated high-level automated vehicles (L3~L5) in more detail.

The 2030 Agenda for Sustainable Development, presented by the United Nations in 2016, encompasses a set of 17 sustainable development goals [2]. The advent of automated vehicles has made a significant contribution to the achievement of these UN goals. Successful integration of automated vehicles into our transportation systems holds immense potential in enhancing road safety [3, 4]. Annually, approximately 1.3 million lives are lost globally due to road accidents, with a substantial proportion comprising children and young adults aged between 5 and 29 years old [5]. By effectively incorporating automated vehicles into our transportation infrastructure, we can substantially reduce injuries and losses caused by traffic accidents. Furthermore, automated vehicles possess the capability to effectively promote sustainable urban development while providing convenient mobility services for individuals facing accessibility challenges [6]. Simultaneously, the widespread adoption of automated vehicles can mitigate issues related to noise pollution and air contamination, thereby making a valuable contribution towards climate action [7].

As the product of new technology, there is a great deal of uncertainty about the attitude of the general public toward automated vehicles. The main source of this uncertainty is popular psychology [8]. To better study the factors affecting this uncertainty, scholars analyzed and discussed the roles of perceived usefulness, perceived ease of use, and perceived risk [9]. In Tang et al.’s study [10], it is pointed out that information dissemination is also an important factor that cannot be ignored. Therefore, further investigation is warranted to explore the public’s inclination toward utilizing automated vehicles in more intricate scenarios. A study conducted by Chen et al. [11] concentrated on gauging the acceptance of fully automated vehicles among the general populace in Australia. The analysis encompassed factors such as perceived ease of use, perceived usefulness, perceived data privacy, perceived trust, attitude, and behavioral intention; revealing their substantial influence on individuals’ attitudes towards adopting automated vehicles.

In a study conducted by Naderi et al. [12], moderating variables such as demographics, psychological characteristics, and driving behavior were employed to examine the impact on the acceptance of automated vehicles. The findings suggest that drivers’ perception of risk associated with hazardous driving behaviors significantly impedes their intention to utilize automated vehicles. Drawing upon the Technology Acceptance Model and the Information System Success Model, Sun [7] developed a new framework for analyzing and discussing the acceptance of automated vehicles. The analysis results demonstrate that perceived ease of use exerts a greater influence on AV acceptance compared to perceived usefulness. The study conducted by Tang et al. [10] introduced the concept of playability into the technology acceptance model to quantitatively measure the impact of psychological factors on individuals’ intention to use automated vehicles. The findings confirm that perceived playability significantly influences users’ intention to adopt AVs, and demographic factors further moderate the influence of psychological factors on this intention.

In summary, the primary objective of this study is to analyze the impact of various factors on the intention to utilize automated vehicles (AVs). Specifically, our study examined the influence of social information, perceived ease of use, perceived usefulness, and perceived risk on intention to use AVs. By investigating respondents’ personality characteristics, we employed an ordered Probit model with random parameters to examine the mediating effect of personality traits on intention to use AVs. The distinct contributions of this paper are as follows:

1. To investigate the factors influencing users’ willingness to use automated vehicles, a questionnaire survey was conducted. An ordered Probit model with random parameters was employed to analyze various factors that may impact the decision, including demographic variables, trust levels, perceived ease of use and usefulness, social information influence, perceived risk perception, and personality characteristics. The findings of this study are valuable for government policymakers and automobile manufacturers in gaining a better understanding of user intentions and developing appropriate strategies to promote the deployment of automated vehicles.
2. This study investigated the mediating role of personality traits in shaping intentions to use automated vehicles, examining how personality characteristics influence social information, perceived ease of use, perceived usefulness, and perceived risk, thereby providing a more comprehensive analysis of factors influencing automated vehicle adoption.
3. The questionnaire designed in this study can be utilized by future researchers to explore users’ intentions regarding automated vehicle usage.

In the subsequent sections of this paper, Section 2 provides a comprehensive review of the existing literature. Section 3 outlines the methodology employed, followed by a result of the model computations in Section 4. Subsequently, Section 5 offers a detailed discussion in response to the obtained results. Finally, Section 6 concludes this study by highlighting its principal findings, limitations, and future research directions.

## 2 Literature review

In recent years, there has been an increasing amount of research into the willingness to use automated vehicles. Scholars have separately looked at socio-demographic attributes [13–15], social information properties [16, 17], perceptual properties [18, 19] and personality traits [13, 20] and other aspects.

### (1) Socio-demographic attributes and their effects

In studies on the willingness to use automated vehicles, most scholars have considered the impact of socio-demographic attributes on willingness to use, mainly: age, gender, education level, and driving experience [9, 10, 21, 22]. Among the studies on age, some of them found that younger groups are more concerned about automated vehicles [23] and have a stronger willingness to use them [24, 25]. However, a study by Charness et al. [13] suggests the opposite, that older age groups are more concerned about and have a stronger desire to use automated vehicles. The study by Jiang et al. [14] also supports this view. The different findings suggest that age is a variable that requires more research to verify its influential role, while the variability in the survey groups across studies may have contributed to the inconsistent results.

Gender shows good consistency in willingness to use. In a study by Charness et al. [13], men were found to be more willing to use automated vehicles than women. This finding was replicated in the study by Payre et al. [26]. This may be because men are more focused on the benefits of automated vehicles, while women have a greater sense of responsibility and show concern [27].

In terms of educational attainment, related studies have shown that people with higher education are more likely to have a higher willingness to use automated vehicles [15, 21]. The reason for this may be that users with higher levels of education are more likely to pursue higher-quality travel [10]. Some studies have found that older drivers with more driving experience are more reluctant to accept the use of automated vehicles, which they believe will impair their enjoyment of driving the vehicle [17, 28, 29].

### (2) Social information properties

Social informativeness refers to the pressure from information that individuals feel when making choices. This effect is especially pronounced when relevant knowledge is weak and external information is oriented in the opposite direction of the choice [10, 30, 31]. In a study by Hegner et al. [32], it was found that negative media coverage about automated vehicles reduces people’s willingness to use automated vehicles. Most of the information people receive when they are concerned about automated vehicles comes from negative media reports, which also tend to cause misjudgment in their choices [16, 32]. And when individuals are considering purchasing a automated vehicle, the opinions of influential people around the individual can also have some influence on their choices [33].

### (3) Perceptual attributes

Perceived usefulness and perceived ease of use are significant influencers in predicting willingness to use [31], and perceived usefulness and perceived ease of use have a significant positive effect on willingness to use automated vehicles [22, 29]. Hegner et al. [32] study pointed out that relevant prior knowledge can improve users’ perceived ease of use and usefulness of automated vehicles, thus increasing people’s willingness to use them. In addition, the results of Zhang et al. showed that perceived ease of use had a higher impact than perceived usefulness [34]. All of these core factors have been shown to have a significant impact on willingness to use [35].

Perceived risk has a significant negative correlation with the intention to use automated driving [36]. People’s lack of knowledge about automated driving technology and perceived external dangers can create a negative attitude and reduce people’s intention to use it [31]. This effect will be especially pronounced when the risks of automated driving are more prominent [19, 37]. However, some scholars have also found that risk perception does not directly affect willingness to use, but indirectly negatively moderates people’s willingness to use [38].

### (4) Personality

An important influence on the willingness to use automated vehicles also comes from the personality of the user. Personality is a stable cognitive, behavioral, and emotional pattern formed by a combination of physiological as well as environmental factors [39]. It has been shown that owners of responsible personalities have a negative intention to use automated vehicles [13]. The more responsible people are, the more likely they are to follow existing rules rather than break them. There is a negative correlation between neuroticism and willingness to use automated vehicle technology [13, 20, 39]. Therefore, personality can be used to predict the willingness of actors to use automated vehicles.

The results indicate that scholars have been gradually studying the intention to use automated vehicles. However, existing studies have mostly focused on the influence of a single dimension, and research on the compound influence of personality and multiple dimensions needs to be deepened. To better investigate the influencing factors of intention to use automated vehicles, this study taps into the key influencing factors of intention to use and the relationship between them based on the heterogeneous differences in individual personality and the above-discussed variables.

## 3 Methodology

At this stage, L3 automated vehicles are not yet available for sale, so the SP questionnaire form was designed to investigate users’ willingness to use them in the future. This section first discusses the survey design used to collect the data, then the reliability test of the questionnaire, and finally the model design.

### 3.1 Methodological review

At this stage, L3 automated vehicles are not yet available for sale, and the choice of using L3 automated vehicles is still in a hypothetical stage, but people’s expectations or concerns may lead to heterogeneity in the outcome of the choice. In other words, any single factor may affect future choices to varying degrees. To analyze this influence, most of the existing studies have used mixed-choice models [14, 15, 25]. The study by Daziano et al. [40] emphasizes that the model should be chosen flexibly for different types of targets. They used different models of logit to estimate the heterogeneity of consumers. Jiang et al. (Jiang et al., 2018) analyzed the ownership of automated vehicles in Japan using a hybrid logit model, using data from 1,728 questionnaires from Japan to analyze to get the influencing factors of automated vehicle ownership. Haboucha et al. [15] used 721 data from Israel and North America to quantify the effects of characteristics on willingness to use and acceptance using a randomized utility model (considering panel effects with logit kernel) considering characteristics of individuals and automated vehicles.

However, the methods described above are not well interpreted as the likelihood of observed factors varying among observations. Therefore, this study will analyze the willingness to use automated vehicles using an ordered probit model with stochastic parameters.

### 3.2 Survey design and data collection

Sojump is an online questionnaire platform, one of the largest survey platforms in China, which is used to collect online survey data [7, 10, 41]. The electronic questionnaire was mainly collected through web links, and the respondents voluntarily participated in filling out the anonymous questionnaire. The formal survey was conducted among 2105 participants aged 18 and above. In the questionnaire, we provided a concise explanation of automated vehicles and the level of automation associated with L3 automated vehicles to the participants: "Automated vehicles employ advanced algorithms and hardware to automatedly perform tasks such as steering control, acceleration, and braking. L3 automated vehicles are capable of transporting you from one location to another without requiring your active involvement in driving. While the vehicle operates automatedly, you can relax, engage with your mobile device, or work on your laptop; however, it is essential that you assume control in case of emergencies."

After the completion of the questionnaire design, three scholars in relevant fields were consulted for preliminary testing. Subsequently, the questionnaire was modified based on their feedback to obtain an initial version. To ensure its quality, three pre-surveys were conducted and adjustments to the questionnaire content were made by collecting feedback, resulting in the final formal version.

The SP method is now widely used to investigate consumers’ willingness to use and preferences for yet-to-be-released products [42, 43]. For this SP survey, five types of attributes were selected based on the literature review of existing studies, and each attribute was set with 4–10 different levels to meet different survey needs. The questionnaire specifically contains four parts. The first section serves as an introduction, encompassing a declaration of data confidentiality and providing a concise overview of the automated vehicle. The second part entails sample characteristics, comprising demographic information such as age, gender, possession of a driver’s license, age at obtaining the license, and education level. The third section encompasses the measured items for six variables: personality [44, 45], social informativeness [46, 47], perceived ease of use [48, 49], perceived usefulness [17, 48], perceived risk [46], and intention to use [48]. The fourth section focuses on participation in automated driving [50] and non-dynamic driving task types.

The pre-survey was conducted three times from the beginning of September 2022 to the end of September 2022, with an interval of 7–9 days. A total of 279, 309, and 293 valid questionnaires were received, respectively. After adjusting the questionnaire according to the pre-survey feedback, the formal survey was conducted from mid-October to mid-December 2022. By comparing the response time of the questionnaire and cross-checking the answers to some questions (for example, obtaining a driver’s license while under the legal age is contradictory), the invalid questionnaires were eliminated, and 1248 valid questionnaires were finally collected. The information of all participants remains completely anonymous throughout the study. The survey item has been officially approved by the College. By local laws and institutional requirements, ethical review and approval are not necessary for this project.

Due to the large period of the dataset, to prove the reliability of the research results. The formal survey was conducted again from November 21, 2023, to December 4, 2023, and 857 valid questionnaires were finally collected.

The information of all participants remains completely anonymous throughout the study. The survey item has been officially approved by the College. In accordance with local laws and institutional requirements, ethical review and approval are not necessary for this project.

**Fig 1:**
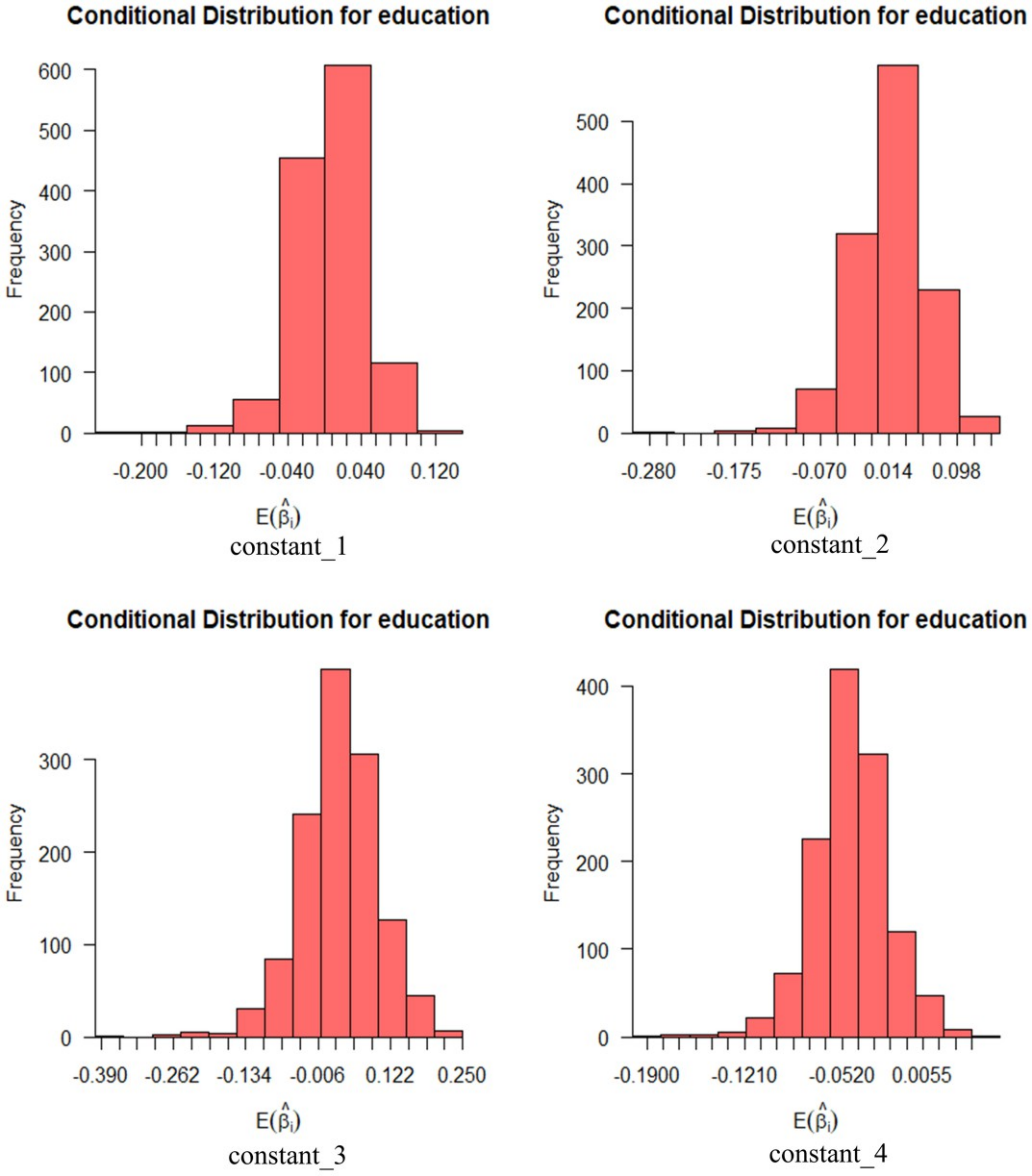
Summary of statistical variables (2022).

A sample of 1,248 people from China participated in the survey. Fig 1 shows the socio-demographic characteristics of the respondents. Of the 1,248 respondents’ data collected, there were 718 male respondents and 530 female respondents, which is more or less equal in terms of male-to-female ratio. In the collected sample data, the age was mostly concentrated in the age group of 26–30 years, which accounted for 38.6% of the total number of the entire respondents, followed by the age group of 31–40 years. The majority of the respondents had a bachelor’s (specialized) degree, which accounted for 49.6% of the total number of respondents, followed by a high school diploma and a postgraduate degree. According to the results of this survey, more than 90% of the respondents have a driver’s license, but their driving experience is on the short side, concentrating on the period of 2~5 years. It is worth noting that close to 80% said they are willing to try using automated vehicles in the future.

**Fig 2:**
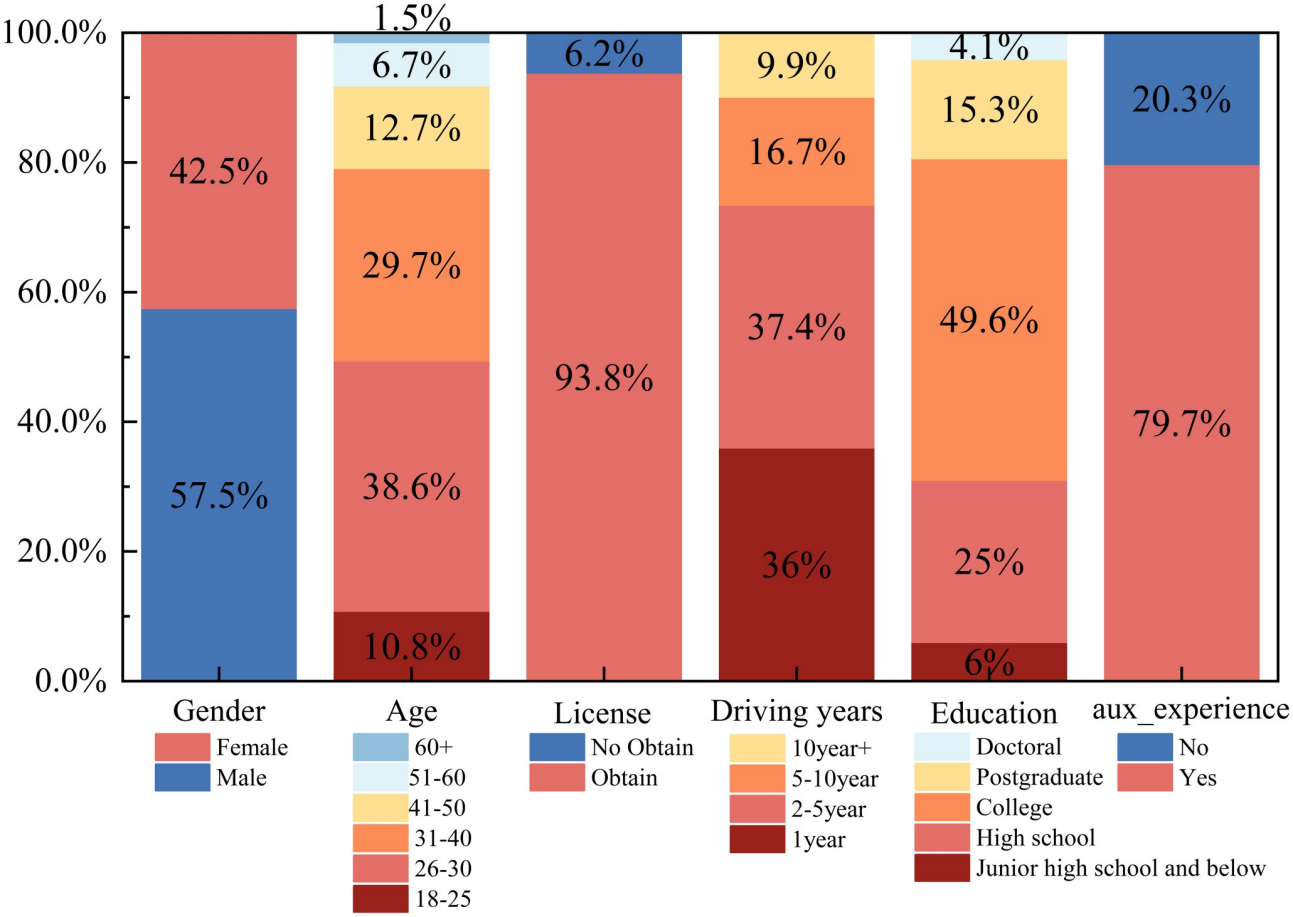
Summary of statistical variables (2023).

For the second survey, up to now, 857 valid questionnaires have been collected. Fig 2 shows the socio-demographic characteristics of the respondents. Of the 857 questionnaires collected, 500 were male respondents and 357 were female respondents, with a roughly equal ratio between men and women. In the collected questionnaires, the age is mainly concentrated in the age group of 26 to 30 years old, with 271 respondents accounting for 31.6% of the total number, followed by the age group of 31 to 40 years old, with 253 respondents accounting for 29.5% of the whole number of respondents. Most of the respondents had college education experience, with 487 accounting for 56.8 percent of the total. More than 90 percent of the respondents had a driver’s license, and more than 70 percent had used a driver assistance system.

In addition, we conducted a reliability test on the collected data, and both Cronbach’s alpha and composite reliability (CR) were above 0.75, and the loadings of the factors were above 0.65. The test results indicate that the data have good usability in terms of internal consistency. The validation results are detailed in S1 and S2 Tables.

### 3.3 Model construction

#### 3.3.1 Problem description

The actor’s willingness to use automated vehicles is influenced by social informativeness, perceived ease of use, perceived usefulness, and perceived risk. Among them, the actor’s adoption of information selectivity is influenced by personality traits. Actors develop their own unique perceived ease of use, perceived usefulness, and perceived risk based on the information they have acquired, and such perceptions will in turn influence willingness to use. Moreover, the change process of actors’ intention to use automated vehicles is unobservable. Therefore, in order to better investigate the change process between actors and the intention to use automated vehicles, an ordered Probit model with random parameters is constructed in this paper to analyze the intention to use.

#### 3.3.2 Model design

*(1) Establishment of potential processes*. As discussed in the previous section, the process of change in willingness to use automated vehicles is unobservable. Moreover, the data obtained from the survey cannot understand the effect of key variables on the change in intention to use automated vehicles. Therefore, this paper assumes that the following potential processes exist for the change in willingness to use. The potential process is described as shown in Eq (1) [51].

$$y_{i}^{*}=\beta_{i}x_{i}+\epsilon_{i};i=1,\cdots ,n;\beta_{i}\sim g\left(\left(\beta_{i}|\theta \right)\right)$$ (1)

where $y_{i}^{*}$ is the final observed result, the *x*_*i*_ is the individual *i* of a set of variables of interest; *β*_*i*_ is the *x*_*i*_ the matrix of coefficients; *ϵ*_*i*_ is the error term for a single respondent; g(β_i_|θ) is the *β*_*i*_ the overall probability density function (PDF). θ is the moments of the distribution, such as the mean and variance.

This paper utilizes *y*_*i*_ as the observed variable and measures the following: very willing (*y*_*i*_ = 4), willing (*y*_*i*_ = 3), neutral (*y*_*i*_ = 2), somewhat reluctant (*y*_*i*_ = 1), and very reluctant (*y*_*i*_ = 0).

*(2) Model construction derivation*. The parameters in the stochastic parameter selection model change in response to changes in the observed values. When the observed variables $y_{i}^{*}$ nature and the overall PDF are known, then the conditional probability density function of the potential process $f\left(y_{i}^{*}|x_{i},\beta_{i}\right)$ is determined. In the present study, the results of $y_{i}^{*}$ have a degree difference between the *ϵ*_*i*_ are logistically distributed, so an ordered choice model is constructed. The PDF of $y_{i}^{*}$ is shown in Eq (2).

$$f\left(y_{i}^{*}|x_{i},\beta_{i}\right)=\prod_{j=1}^{J}{\left[F\left(k_{j}-\beta_{i}x_{i}\right)-F\left(k_{j-1}-\beta_{i}x_{i}\right)\right]}^{y_{i}},m=1,\cdots \cdots ,M$$ (2)

Where *F*(.) represents the cumulative distribution function (cdf) of error terms. In the ordered model, *k*_*j*_ represents the alternative threshold of *j* = 1, ⋯, *J*, such that *k*_0_ = −∞ or *k*_*J*_ = +∞.

In the potential process, the coefficients of each actor *β*_*i*_ is different from each other. Therefore, assuming that *β*_*i*_ that each element in is independently normally distributed, the *β*_*i*_ the first element of the *k* element is $\beta_{k}\sim N\left(\beta_{k},\sigma_{k}^{2}\right)$. Each specific coefficient can be written as Eq (3).


$$\beta_{ki}=\beta_{k}+\sigma_{k}\omega_{i},\omega_{i}\sim N\left(0,1\right)$$ (3)

In Eq (3), the heterogeneity of individual *i* is captured by the standard deviation *σ*_*k*_. Therefore, when *σ*_*k*_ = 0, then the model is simplified to a fixed parameter model; when *σ*_*k*_ > 0, *β*_*k*_ will serve as a marginal effect of the potential dependent variable unique to the individual. In other words, the *β*_*i*_ There is a heterogeneity in the characteristics that make *β*_*i*_ the effect on *y*_*i*_ different effects to occur. However, the *β*_*i*_ is not observable, and we cannot know how this effect varies between individuals. In order to observe *β*_*i*_, one can use Eq (4) to calculate the distribution parameters θ of the conditional probabilities obtained.


$$P_{i}\left(\theta \right)=\int_{\beta_{i}}^{0}P\left(y_{i}|x_{i},\beta_{i}\right)g\left(\beta_{i}\right)d\beta_{i}$$ (4)

In Eq (4), there is no approximate solution for the probability, which makes it difficult to integrate the stochastic parameters and thus to perform the maximum likelihood (ML) estimation. To solve this problem, Sarrias et al. [51] scholars pointed out that Monte Carlo Integration (MCI) can be a good solution to this problem. This is shown in Eq (5).

$${\tilde{P}}_{i}=\frac{1}{R}\sum_{r=1}^{R}\tilde{P}\left(y_{i}|x_{i},\beta_{ir}\right)$$ (5)

Where $\sum_{r=1}^{R}\tilde{P}\left(y_{i}|x_{i},\beta_{ir}\right)$ is the simulated probability for individual respondent *i*, evaluated at the r-th iteration of *β*_*i*_, and *R* is the total iteration numbers. Assuming that when the choices of all respondents are independent, the estimated value of simulated maximum likelihood (SML) θ satisfies Eq (6).


$${\hat{\theta }}_{SML}=\text{arg}\;{\text{max}}_{\theta \in \Theta }\sum_{i=1}^{N}\text{log}\;{\tilde{P}}_{i}\left(\theta \right)$$ (6)

*(3) Variable selection*. In this model, respondents’ willingness to use automated vehicles was used as the dependent variable for the study. For the exploratory variables (independent variables), the following variables were used to build the final model: 1) gender, 2) age, 3) driver’s license, 4) personality, 5) education, and 6) experience of assisted driving use. Among the variables for the age group, we removed the age group under 18 years old, considering the regulations for obtaining a driver’s license in China. Among the independent variables, certain variables shall be random parameters if their standard deviations are statistically significant within a normal distribution. Therefore, the following variables were set as random parameters in this study: 1) age, and 2) education. In our analysis, we processed the results of social informativeness and perceived risk. In this case, the higher the score of social informativeness, the higher the influence of the bright side of social informativeness. Higher scores on perceived risk represent a lower level of perceived risk for the respondent.

## 4 Results

### 4.1 Willingness to use

The results of the ordered Probit model with random parameters are shown in S3 Table. Kappa.1, Kappa.2, and Kappa.3 in S1 Table represent the thresholds of the model. Of the three thresholds, constant_2(more frequent trips with automated vehicles) has the highest, except kappa.3. This means that respondents’ willingness to use automated vehicles for travel is more likely to be a long-term use.

In the results of the analysis, gender did not appear to be statistically significant, suggesting that gender is not a key influencing factor in driving usage intentions. Possession of a driver’s license was statistically significant, and possession of a driver’s license showed negative signs in the willingness to use automated vehicles (constant_1: *β*_1_ = −0.381, *z*_1_ = −2.786, *p*_1_ < 0.01; constant_3: *β*_3_ = −0.251, *z*_3_ = −1.815, *p*_3_ < 0.1). Age showed negative statistical significance (constant_1: *β*_1_ = −0.075, *z*_1_ = −2.520, *p*_1_ < 0.05; constant_2: *β*_2_ = −0.066, *z*_2_ = −2.230, *p*_2_ < 0.05; constant_3: *β*_3_ = −0.068, *z*_3_ = −2.265, *p*_3_ < 0.05). The utilization of a driver assistance system demonstrates an adverse effect on constant_1 (when employing an automated vehicle) (constant_1: *β*_1_ = −0.200, *z*_1_ = −3.012, *p*_1_ < 0.01).

Extraversion has a positive significance on constant_3(recommending a automated vehicle to friends around you) (constant_3: *β*_3_ = 0.045, *z*_3_ = 1.681, *p*_3_ < 0.1). Conscientiousness showed negative signs on constant_3(recommending automated vehicles to friends around you) (constant_3: *β*_3_ = −0.065, *z*_3_ = 1.681, *p*_3_ < 0.05). Neuroticism showed positive effects on constant_2(frequent use of automated vehicles) (constant_2: *β*_2_ = 0.050, *z*_2_ = 1.855, *p*_2_ < 0.1).

Among the three levels of social information, perceived ease of use, perceived usefulness, and perceived risk, the threshold of perceived usefulness is the highest. The threshold for risk perception was lowest, except at the constant_2(frequent use of automated vehicles) level.

**Fig 3:**
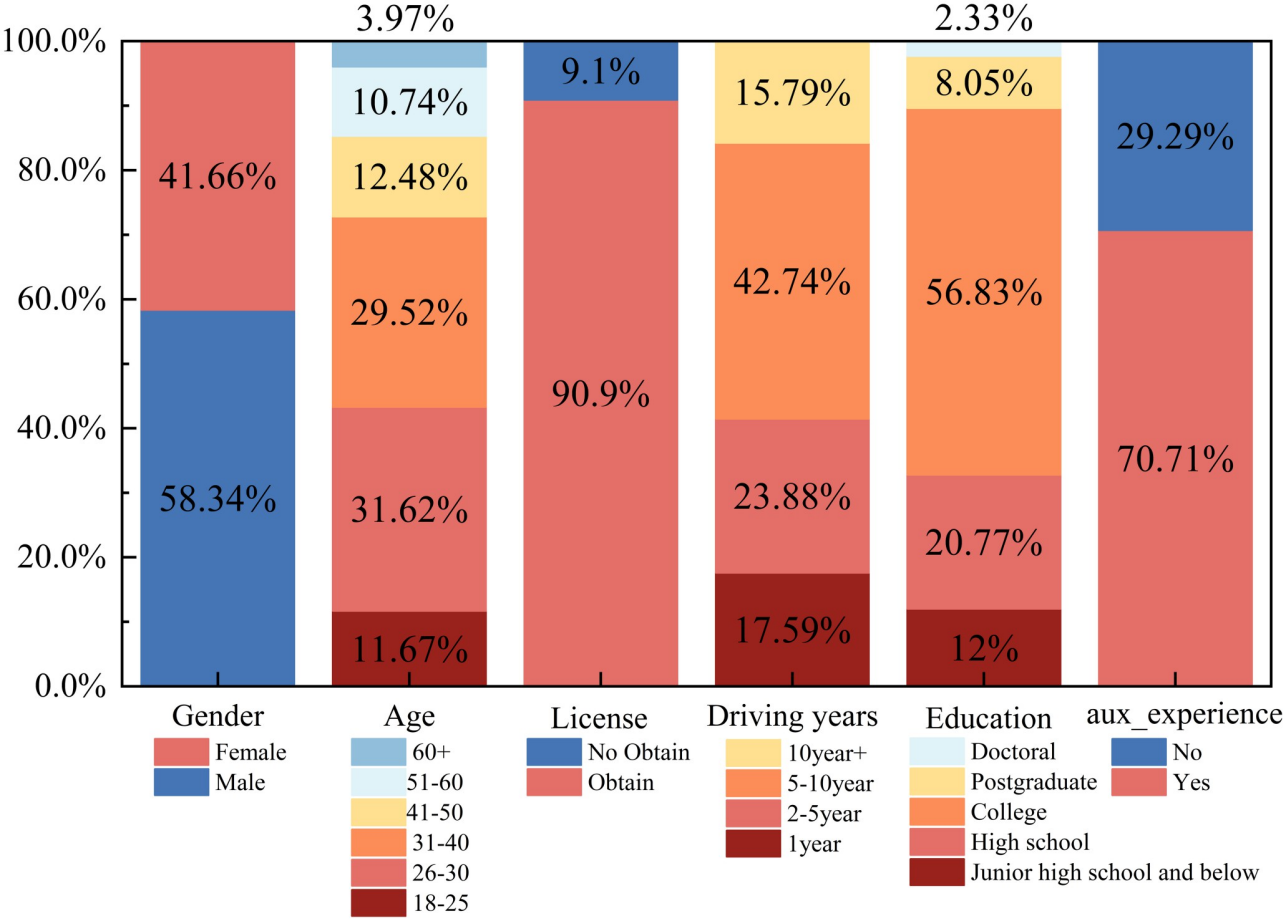
Conditional mean distribution of willingness to use random variables (age (top), education level (bottom)).

As mentioned above, the following two variables are set as random parameters: 1) age and 2) highest level of education. The standard deviations of the two are not statistically significant, indicating that their effects on the intention to use are generally the same or similar. Fig 3 shows the influence distribution of random parameters on different levels of usage intention, with a probability interval of 95%. Among the individuals who are willing to use automated vehicles, the expectation coefficient value distribution of the highest education level is wide, but the expectation value distribution of age is narrow. And the condition distribution of different levels of use intention of different individuals is also different.

### 4.2 Social informativeness

The estimation results of social informativeness are shown in S4 Table. The thresholds in S2 Table show that, except for kappa.1, constant_4 (obtaining information about automated vehicles through social media) has the highest threshold. This means that people believe that information obtained from social media is more authentic and reliable and better reflects the advantages and disadvantages of the new technology.

The coefficient values of the variables show the influence of various factors on social informativeness. Gender does not seem to be statistically significant in the model of social informativeness, while possession of a driver’s license is only statistically significant at a single level (constant_4: *β*_4_ = 0.335, *z*_4_ = 2.473, *p*_4_ < 0.05). Age was statistically negatively significant (constant_1: *β*_1_ = −0.115, *z*_1_ = −4.031, *p*_1_ < 0.001; constant_2: *β*_2_ = −0.124, *z*_2_ = −4.170, *p*_2_ < 0.001; constant_3: *β*_3_ = −0.144, *z*_3_ = −4.771, *p*_3_ < 0.001; constant_4: *β*_4_ = −0.125, *z*_4_ = −4.187, *p*_4_ < 0.001). The personality variables openness (constant_2: *β*_2_ = 0.081, *z*_2_ = 2.787, *p*_2_ < 0.01; constant_4: *β*_4_ = 0.082, *z*_4_ = 2.876, *p*_4_ < 0.01) and neuroticism (constant_4: *β*_4_ = 0.055, *z*_4_ = 2.002, *p*_4_ < 0.05) were statistically significant and showed positive effects.

The following is an exploratory analysis of the variables of age and education. Generally, when the standard deviation of the independent variable is statistically significant, then its effect on the dependent variable is differentiated in general. The standard deviation of age is not statistically significant, whereas educational attainment is. Of course, educational attainment is not statistically significant at every level (constant_2: *β*_2_ = 0.081, *z*_2_ = 2.922, *p*_2_ < 0.01; constant_3: *β*_3_ = 0.108, *z*_3_ = 3.892, *p*_3_ < 0.001; constant_4: *β*_4_ = 0.092, *z*_4_ = 3.236, *p*_4_ < 0.01).

**Fig 4:**
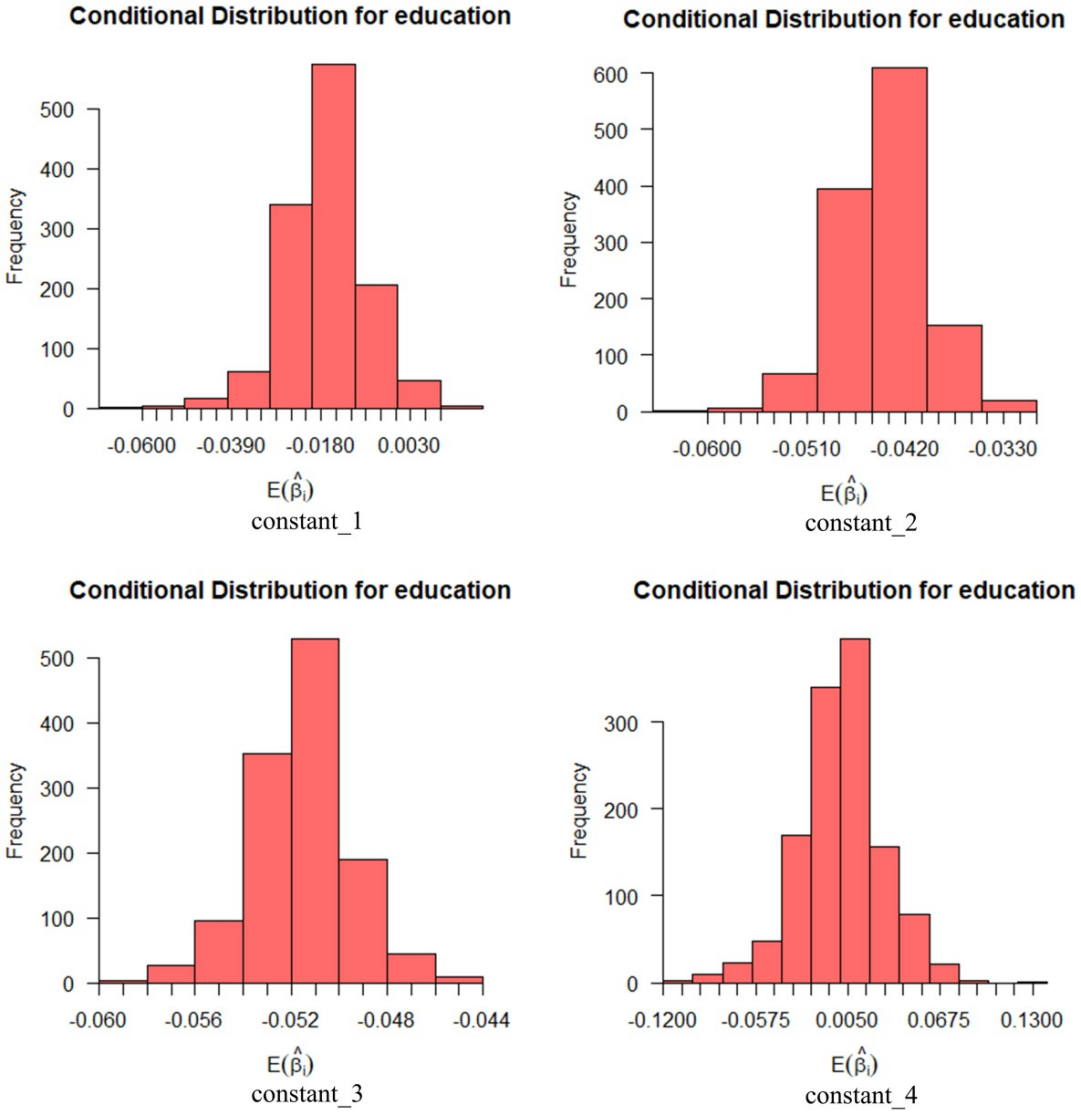
Conditional mean distribution of socially informative age variables.

**Fig 5:**
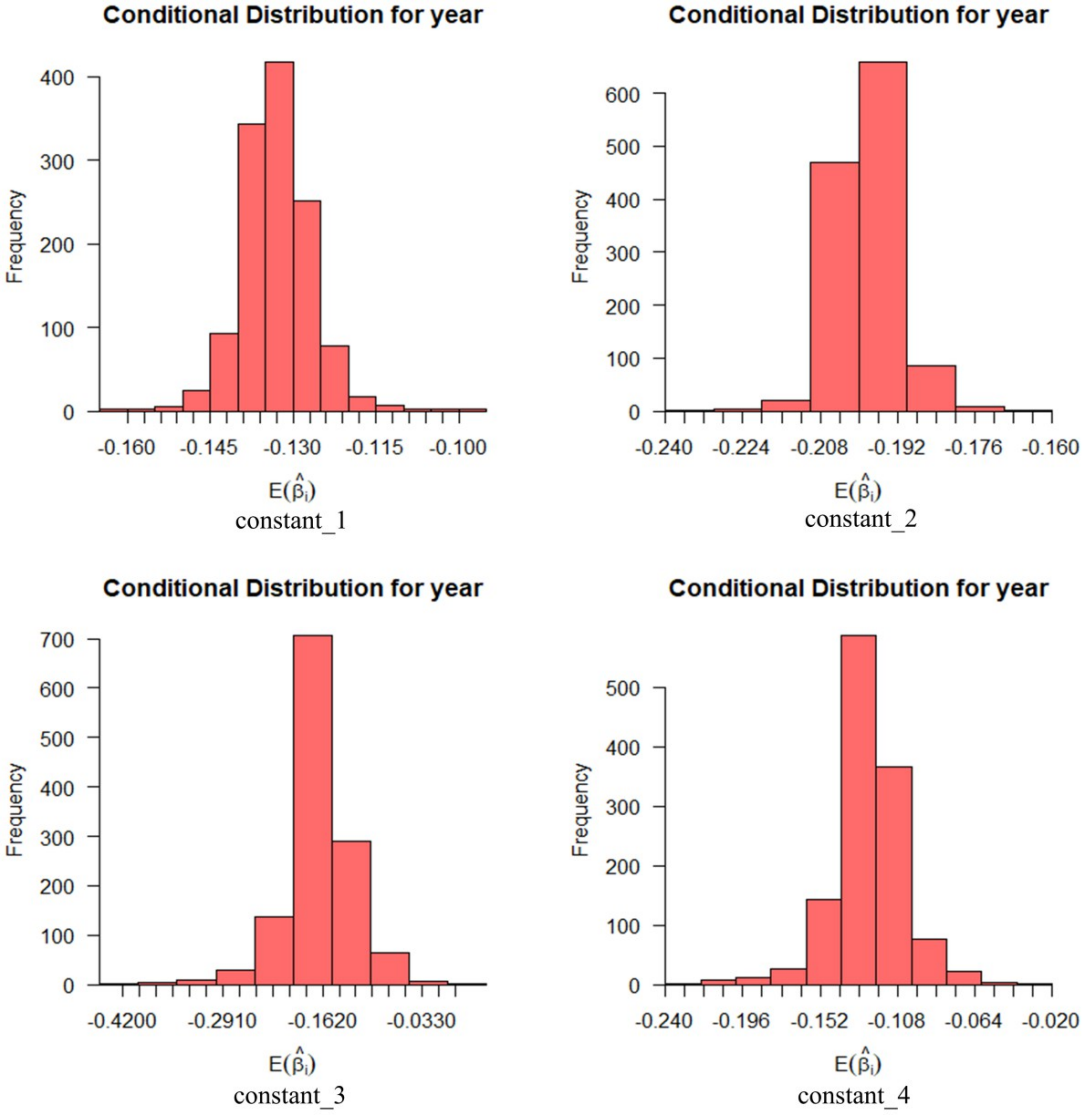
Conditional mean distribution of socio-informative educational attainment variables.

Figs 4 and 5 show the effect of these random variables on social informativeness with a 95% probability interval. In the figures, we can find that the expectation coefficient for age is more widely distributed at the levels of news media and relevant car companies, while education is more widely distributed at the levels of friend referrals and social media.

### 4.3 Perceived ease of use

The result estimates of perceived ease of use are shown in S5 Table. Except for kappa.3, perceived ease of use has the highest threshold for constant_4 (process memorability), indicating that the complexity of the operational process will directly affect people’s perceived ease of use.

Gender is statistically significant at only one level (constant_1: *β*_1_ = −0.125, *z*_1_ = −1.980, *p*_1_ < 0.05) in the model of perceived ease of use. Agreeableness was statistically significant but showed a negative effect (constant_2: *β*_2_ = −0.055, *z*_2_ = −1.855, *p*_2_ < 0.1; constant_3: *β*_3_ = −0.052, *z*_3_ = −1.836, *p*_3_ < 0.1; constant_4: *β*_4_ = −0.060, *z*_4_ = −2.012, *p*_4_ < 0.05). Openness is also statistically significant and has a positive and positive effect on perceived ease of use (constant_3: *β*_3_ = 0.064, *z*_3_ = 2.319, *p*_3_ < 0.05; constant_4: *β*_4_ = 0.061, *z*_4_ = 2.119, *p*_4_ < 0.05). Age is statistically significant and exhibits a negative effect (constant_2: *β*_2_ = −0.064, *z*_2_ = −2.159, *p*_2_ < 0.05; constant_3: *β*_3_ = −0.067, *z*_3_ = −2.358, *p*_3_ < 0.05; constant_4: *β*_4_ = −0.051, *z*_4_ = −1.686, p_4_ < 0.1).

Exploratory analyses were conducted for two random parameters of age, and education. The standard deviation of age was statistically significant, indicating that the effect of age on perceived ease of use was differentiated overall (constant_1: *β*_1_ = 0.096, *z*_1_ = 2.405, *p*_1_ < 0.05; constant_2: *β*_2_ = 0.088, *z*_2_ = 1.973, *p*_2_ < 0.05; constant_4: *β*_4_ = 0.084, *z*_4_ = 2.122, *p*_4_ < 0.05). Only one dimension of education was statistically significant, indicating that the effect of age on perceived ease of use was more significant than education (constant_4: *β*_4_ = 0.083, *z*_4_ = 2.053, *p*_4_ < 0.05).

**Fig 6:**
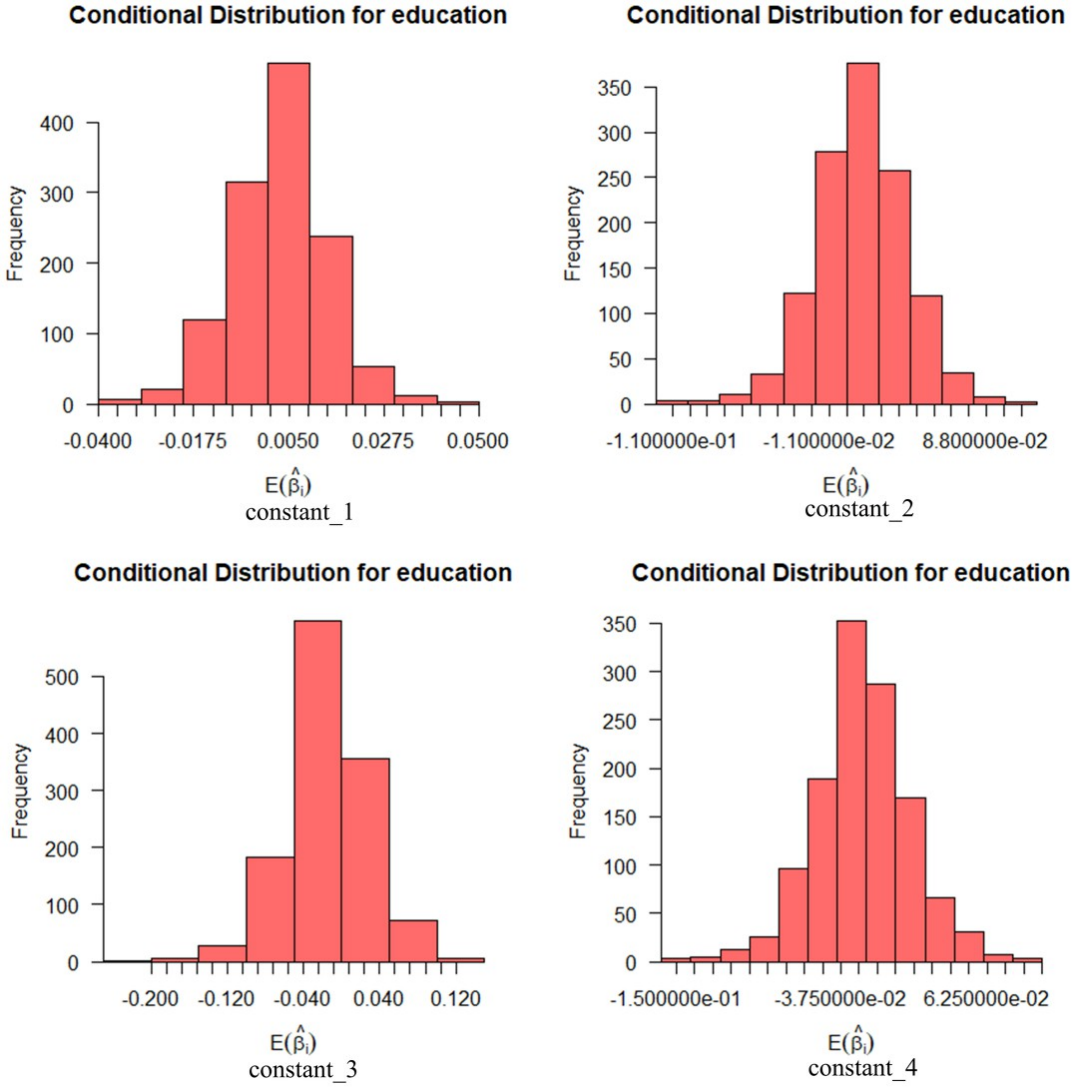
Conditional mean distribution of perceived ease of use age variables.

**Fig 7:**
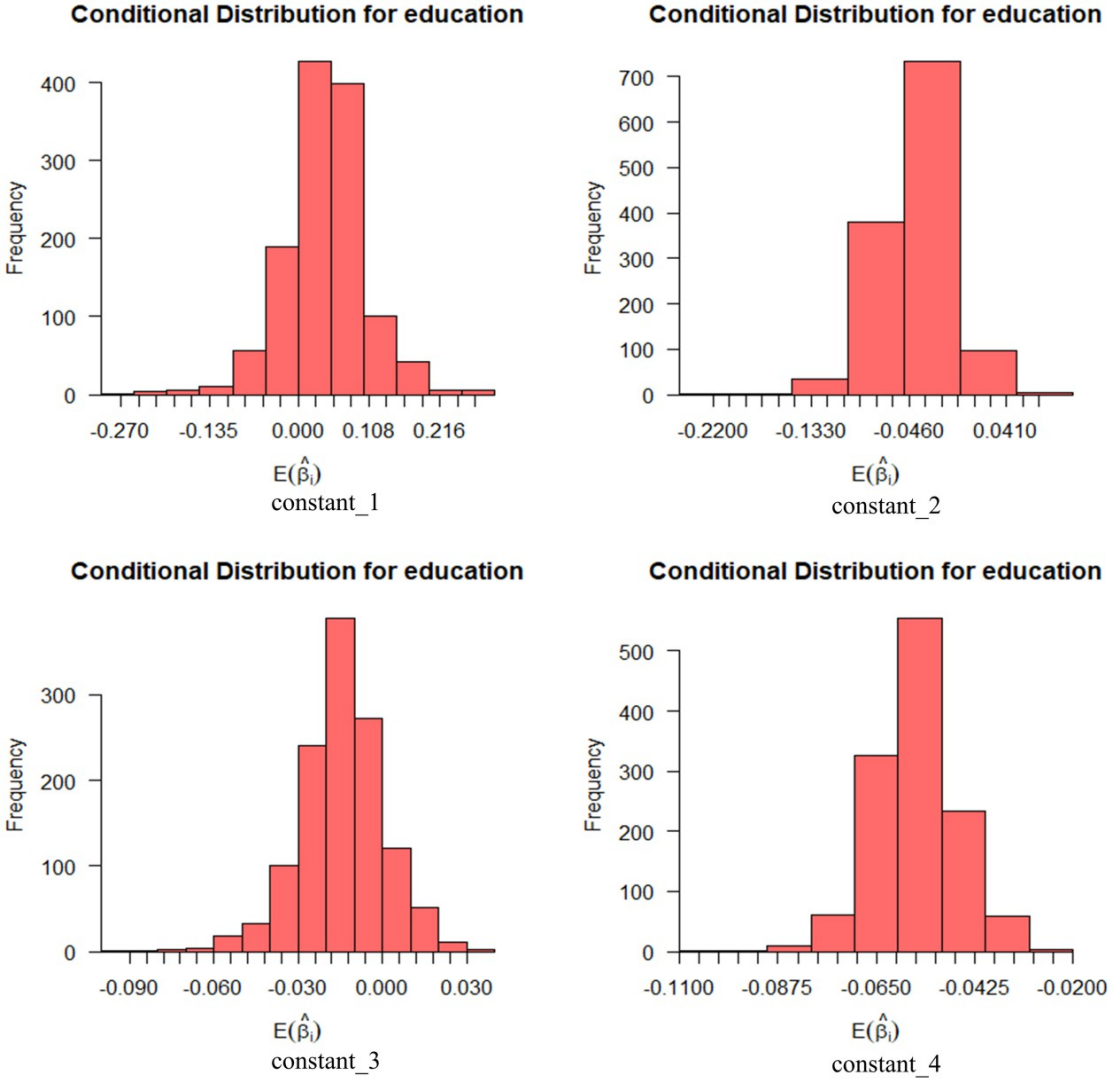
Conditional mean distribution of perceived ease of use education level variables.

Figs 6 and 7, show the effect of these variables on perceived ease of use with a 95% probability interval. Age has the widest distribution of expectation coefficients in operational processes. Education has the widest distribution of expectation coefficients on the remaining three levels.

### 4.4 Perceived usefulness

The estimation results of perceived usefulness are shown in S6 Table. Among the perceived usefulness, constant_1 (more time to do other things in autopilot) has the highest threshold and cosntant_4 (lower travel costs) has the lowest.

Age was statistically significant but presented a negative effect (constant_1: *β*_1_ = −0.098, *z*_1_ = −3.148, *p*_1_ < 0.05; constant_2: *β*_2_ = −0.133, *z*_2_ = −4.259, *p*_2_ < 0.001; constant_3: *β*_3_ = −0.084, *z*_3_ = −2.753, *p*_3_ < 0.05; constant_4: *β*_4_ = −0.112, *z*_4_ = −3.811, *p*_4_ < 0.001). Openness to personality showed positive affectivity in the statistics of perceived usefulness and was statistically significant (constant_1: *β*_1_ = 0.066, *z*_1_ = 2.205, *p*_1_ < 0.05; constant_4: *β*_4_ = 0.047, *z*_4_ = 1.696, *p*_4_ < 0.1).

The analysis was conducted for the two random variables of age (constant_1: *β*_1_ = 0.076, *z*_1_ = 2.219, *p*_1_ < 0.05; constant_2: *β*_2_ = 0.089, *z*_2_ = 2.271, *p*_2_ < 0.05; constant_3: *β*_3_ = 0.100, *z*_3_ = 2.898, *p*_3_ < 0.01; constant_4: *β*_4_ = 0.078, *z*_4_ = 2.075, *p*_4_ < 0.05) and education (constant_1: *β*_1_ = 0.138, *z*_1_ = 4.298, *p*_1_ < 0.001; constant_2: *β*_2_ = 0.086, *z*_2_ = 2.517, *p*_2_ < 0.05) in perceived usefulness. The standard deviation of both age and education is statistically significant. This indicates that the impact of education and age on perceived usefulness is overall different from each other.

**Fig 8:**
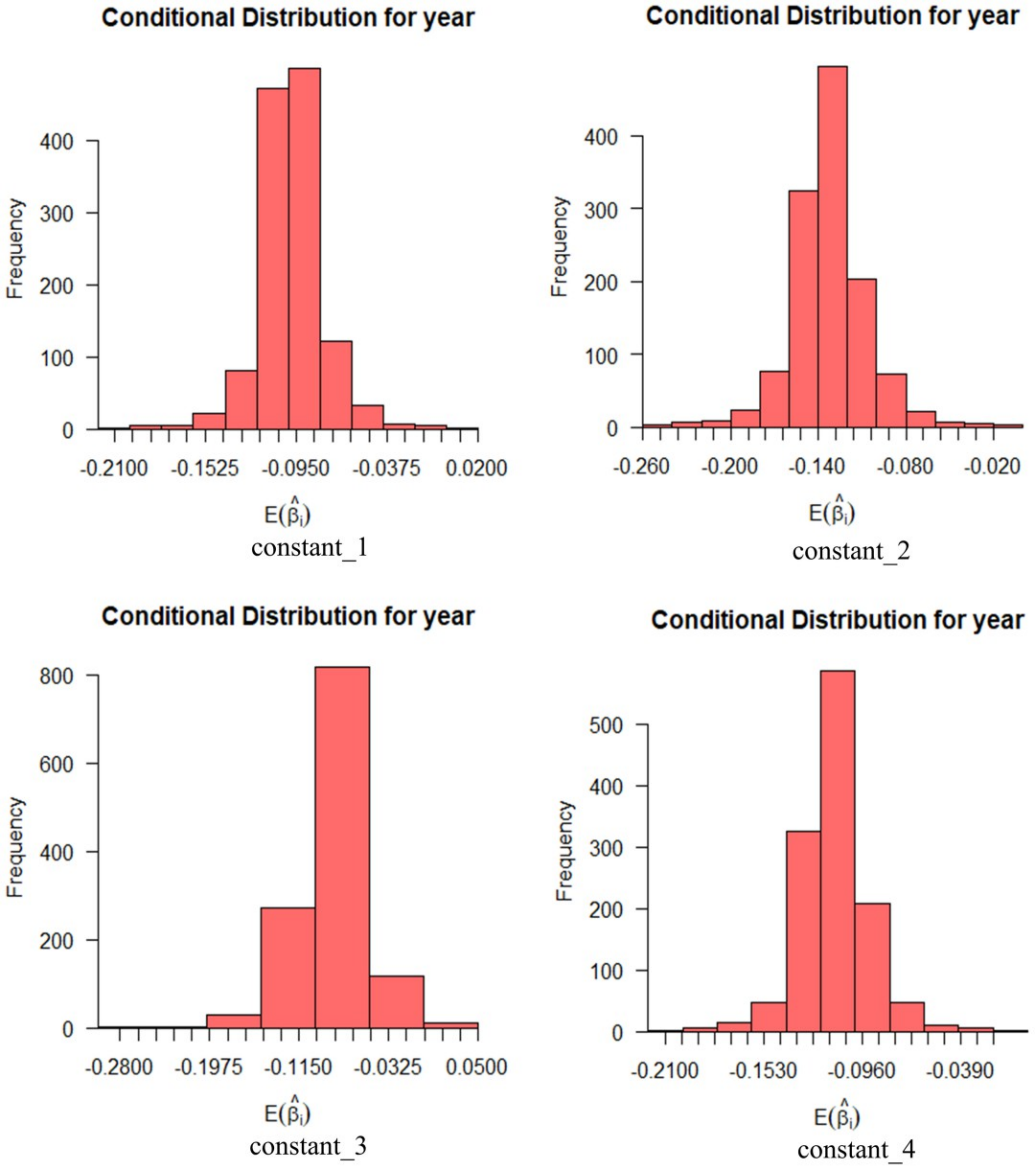
Conditional mean distribution of perceived usefulness age variables.

**Fig 9:**
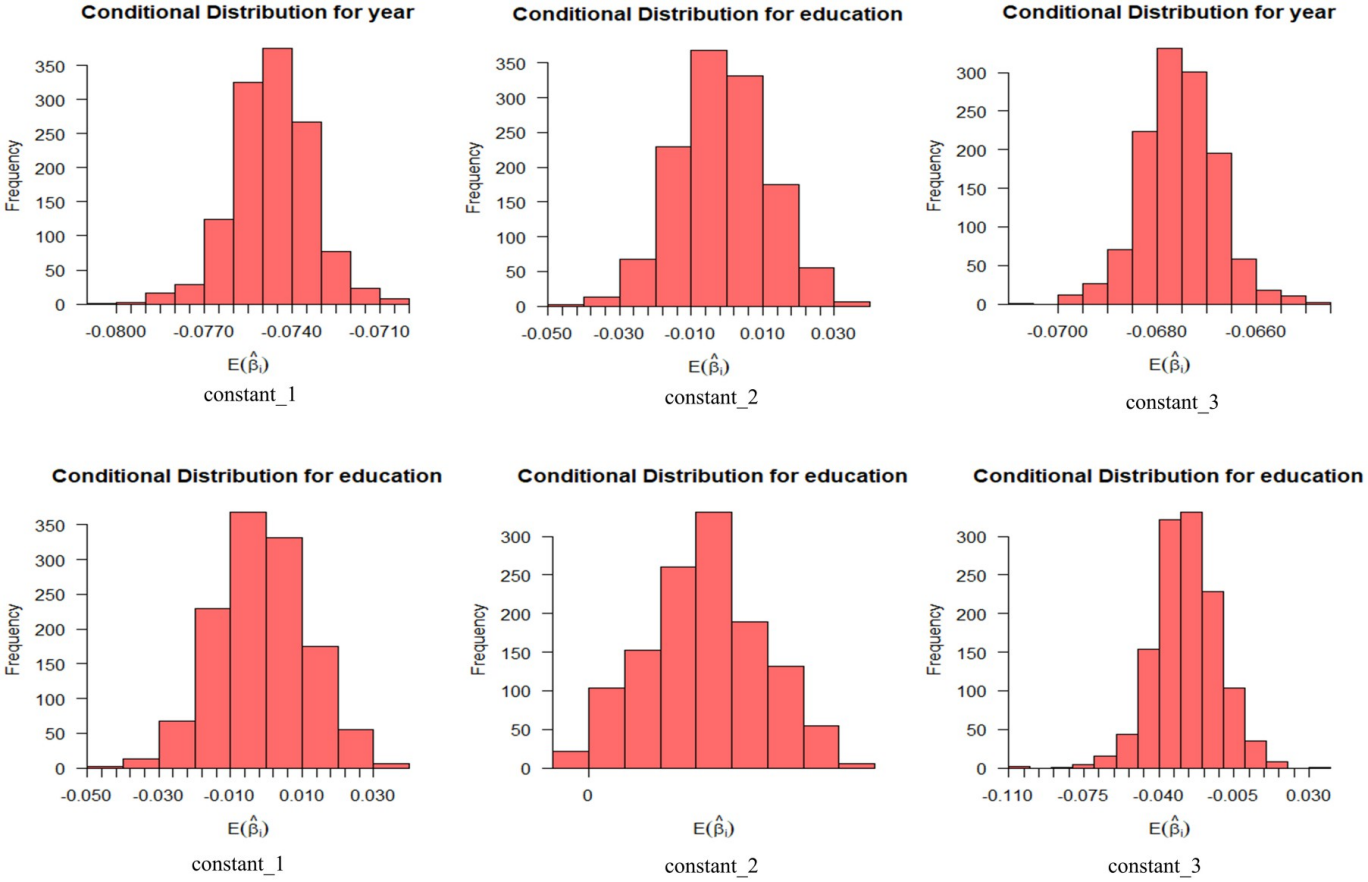
Conditional mean distribution of perceived usefulness education level variables.

Figs 8 and 9 show the effect of age and education on perceived usefulness with a 95% probability interval. Age has the widest distribution of expectation coefficients at both the travel ability and travel cost levels, while education has the widest distribution at both time use and travel efficiency.

### 4.5 Perceived risk

S7 Table shows the estimated results of risk perception. In the threshold of risk perception, except kappa.2, constant_3(privacy security) has the highest threshold, while constant_4(danger caused by misoperation) has the lowest threshold.

Gender was statistically significant (constant_1: *β*_1_ = −0.141, *z*_1_ = −2.183, *p*_1_ < 0.05; constant_2: *β*_2_ = −0.112, *z*_2_ = −1.671, *p*_2_ < 0.1), except for constant_3(privacy security) and constant_4(risk of operational error). In the analysis of personality, extraversion (constant_3: *β*_3_ = 0.054, *z*_3_ = 1.772, *p*_3_ < 0.1), conscientiousness (constant_3: *β*_3_ = 0.054, *z*_3_ = 1.733, *p*_3_ < 0.1), and neuroticism (constant_3: *β*_3_ = 0.068, *z*_3_ = 2.185, *p*_3_ < 0.05) were statistically significant on constant_3 (privacy security) and showed positive effects. However, agreeableness has a negative significance on constant_2 (equipment security risk) (constant_2: *β*_2_ = −0.053, *z*_2_ = −1.744, *p*_2_ < 0.1). Openness has a positive effect on constant_1 (environmental adaptation) (constant_1: *β*_1_ = 0.065, *z*_1_ = 2.251, *p*_1_ < 0.05).

Two random variables of risk perception, age(constant_3: *β*_3_ = 0.121, *z*_3_ = 3.404, *p*_3_ < 0.001), and education background(constant_1: *β*_1_ = 0.097, *z*_1_ = 2.647, *p*_1_ < 0.01; constant_2: *β*_2_ = 0.114, *z*_2_ = 3.155, *p*_2_ < 0.01; constant_3: *β*_3_ = 0.155, *z*_3_ = 4.513, *p*_3_ < 0.001; constant_4: *β*_4_ = 0.080, *z*_4_ = 1.915, *p*_4_ < 0.1), were analyzed. The standard deviations of age and education are statistically significant, which means that the effects of age and education on risk perception are generally different.

**Fig 10:**
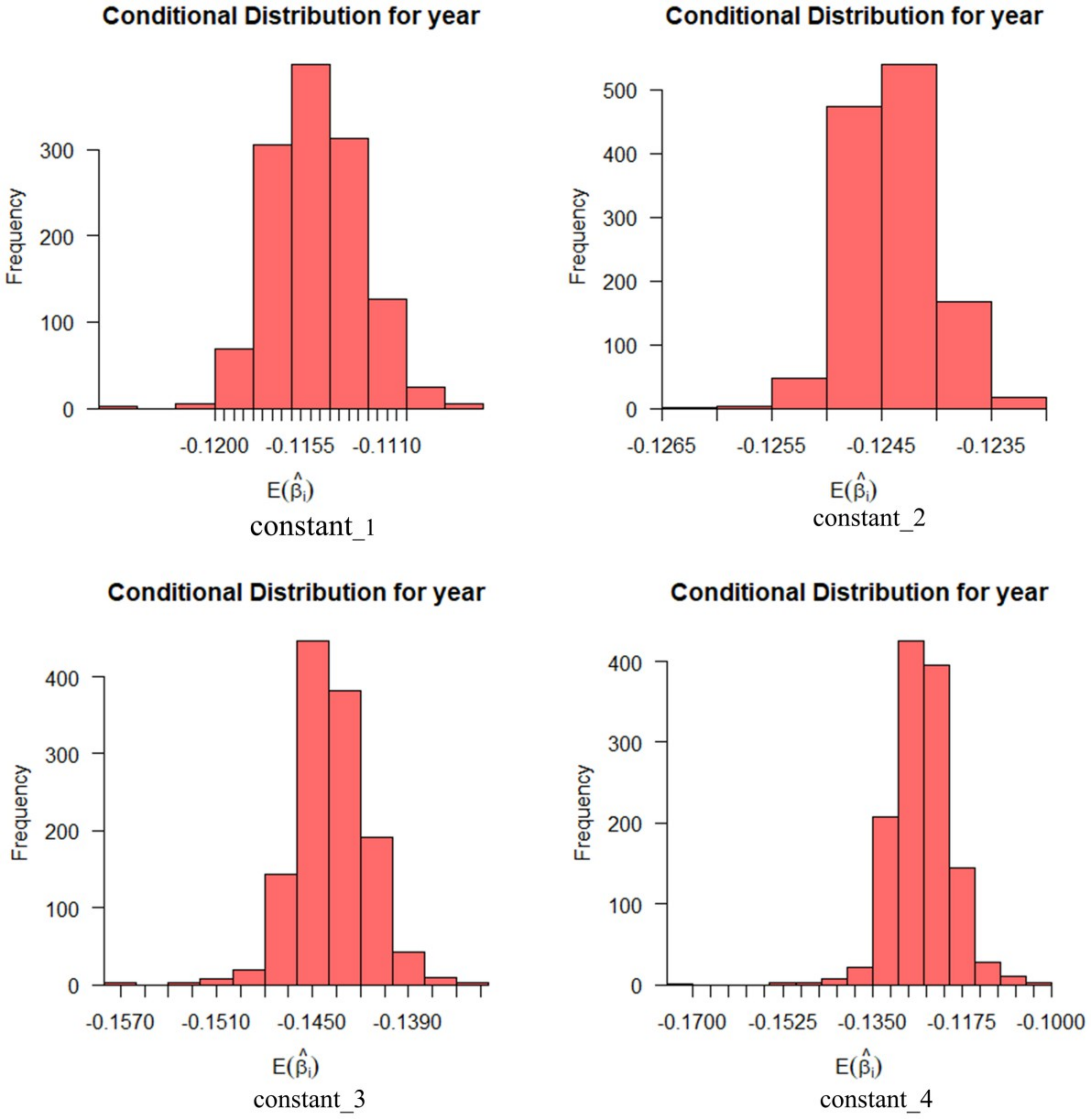
Conditional mean distribution of risk perceptibility age.

**Fig 11:**
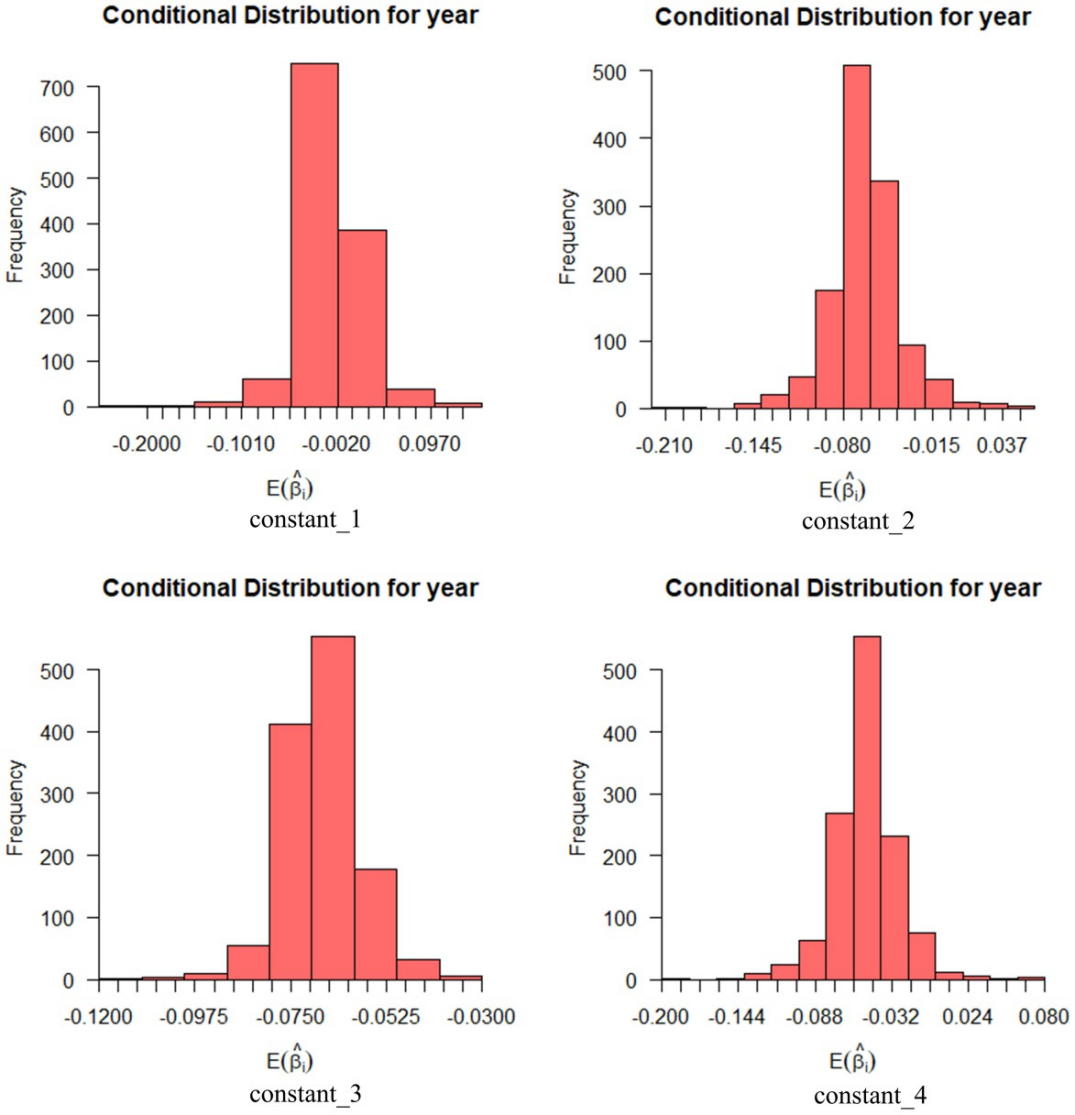
Conditional mean distribution of perceived risk education level.

Figs 10 and 11 show the effect of age and education on risk perception with a probability interval of 95%. Education has the widest distribution of the expectation coefficient in privacy security and operation security, and age has the widest distribution of the expectation coefficient in environmental adaptability.

## 5 Discussion and implications

### 5.1 Discussion

The ability of individuals to derive benefits from new technology is a prerequisite for their acceptance and utilization, which holds for AVs as well. In this study, the collected data was analyzed using an ordered Probit model with random parameters. The findings indicate that social information, perceived ease of use, perceived usefulness, and perceived risk significantly influence the intention to use AVs in this research. The perceived usefulness can exert a stronger influence on individuals’ intention to utilize Avs (*β*_2022_ = 0.082, *z*_2022_ = 8.423, *p*_2022_ < 0.001, *β*_2023_ = 0.084, *z*_2023_ = 5.692, *p*_2023_ < 0.001). Xiao et al. [52] used data from the 2019 California Vehicle Survey to analyze the decision-making process of people’s willingness to use autonomous vehicles. The finding that perceived usefulness is an essential factor in determining willingness to use is consistent with our findings. Through mediation analysis, this study found that personality plays a mediating role in the intention to use Avs. This result is consistent with that of Nordhoff et al. [53].

As anticipated, negative social information exerts a detrimental influence on the intention to use AVs, aligning with previous research findings. For instance, Du et al. [54] discovered that media reports about AVs significantly impact users’ usage intentions, with negative reports exhibiting a negative correlation. This can be attributed to the fact that adverse media coverage of AVs leads to user misconceptions and inappropriate reactions [55]. Given that most individuals have yet to experience AVs firsthand, their knowledge primarily stems from media reports. Positive media portrayal of AVs effectively enhances self-efficacy and fosters public willingness toward their adoption [12]. Overall, these findings underscore the significance of social information in shaping intentions regarding AV usage.

In addition, it was found that perceived usefulness more strongly predicted the intention to use AVs compared to perceived ease of use, which is consistent with the conclusions of previous studies [10, 34, 56, 57]. In the study of Zhang et al. [22], it was pointed out that perceived ease of use and perceived usefulness were proved to have a significant impact on AV usage intention. This is because perceived ease of use and perceived usefulness provide people with perceived value about AVs, which are composed of perceived benefits and perceived costs. The perceived usefulness of AVs is considered to directly improve the convenience of their lives [31] and represents a vested interest of people. Perceived ease of use represents the subjective tendency of people to perceive the difficulty of operating AVs [58], which is a kind of invisible cost. Therefore, vehicle manufacturers can maximize AVs acceptance by informing users about the received benefits of using AVs (practicality) and at the same time informing existing users how to effectively and conveniently use AVs ease of use.

The perceived risk associated with new technology poses a significant impediment to the advancement of new technology. In other words, an excessively high perception of risk can impede individuals’ inclination to utilize AVs. This is primarily attributed to the fact that an exaggerated perception of risk can undermine the driver’s ability to operate AVs effectively, subsequently diminishing users’ intention to adopt them. Deng et al. [59] study corroborates our findings.

We also carefully surveyed respondents who were reluctant to use AVs. The data showed that 180 participants expressed concerns about the safety of autonomous vehicles in extreme environments, while 160 respondents expressed doubts about their overall safety. In addition, 90 respondents highlighted the importance of strengthening regulations related to AVs. This means that people’s safety perception of AVs is the primary factor affecting people’s use of AVS [60]. The observations of this study are consistent with existing research that security plays a crucial role for people to use AVs. Moreover, one of the main reasons people worry about system security is the leakage of private data. This finding is consistent with the conclusion of the study by Chen et al. [11] that private data can significantly affect the intention to use AVs. Our results show that the perfection of regulations is also one of the factors hindering people’s intention to use autonomous vehicles. They fear that an improper division of responsibility in an accident would infringe on their interests. The above findings indicate the importance of risk perception on AV use intention, which will influence people’s adoption of AVs.

Many scholars have emphasized that the influence of personality traits should be considered when predicting automatic trust and acceptance, but the role of personality in the acceptance of willingness to use has been less confirmed than other research factors [61, 62]. Our study makes several contributions to this. Personality was found to mediate the effect of intention to use. Openness and extraversion were significant predictors of perceived ease of use and usefulness. Openness and extraversion increase people’s intention to use AVs by influencing perceived ease of use and perceived usefulness. People with open and extroverted personalities are curious about the emergence of new technologies and are more likely to feel the availability and ease of use of new technologies [62, 63].

Surprisingly, neuroticism has a positive effect on the intention to use, contrary to the conclusions of previous studies [13, 20]. Previous research has shown that neuroticism is at the heart of "emotional variability." However, Kalokerinos et al. [64] recently published an article in PNAS suggesting the opposite conclusion, that the typical mood of highly neurotic people is characterized by a constant and stable low mood rather than a variable mood. Neuroticism is more sensitive to negative than positive information [65–67], thus neuroticism can be understood as a person with negative prior beliefs. When the results proved favorable, there was some improvement in neural core beliefs.

There are also some interesting findings that people with driver’s licenses are more reluctant to use automated vehicles. It may be that automated vehicles impair their enjoyment of driving, leading to their negative attitude towards using automated vehicles [22]. Respondents with experience with driver assistance systems showed resistance to automated vehicles. This means that the driver assistance system failed to reach the psychological expectations of the respondents and reduced the perceived usefulness of the respondents.

To address the time limitation of the sample, a new survey was conducted in November 2023. Upon analyzing the survey results, it was observed that social information, perceived ease of use, perceived usefulness, and perceived risk continue to be the primary factors influencing individuals’ intention to use automated vehicles. Furthermore, this investigation revealed a stronger mediating effect of personality on AV usage intention. Openness and extraversion were found to positively impact social information, perceived usefulness, perceived ease of use, and perceived risk—aligning with our previous findings. This further substantiates the validity of our research conclusions.

### 5.2 Implications

The implications of this study pertain to both theoretical and practical aspects, providing valuable insights for government policy makers and automobile manufacturers in promoting automated vehicles. In this study, an ordered Probit model with random parameters was employed to analyze the influencing factors on the intention to use automated driving. By incorporating personality variables into the analysis of intention towards automated vehicles, it is possible to enhance external validity by examining the mediating effect of personality. Furthermore, parameter randomization determining thresholds can effectively elucidate individual heterogeneity in behavioral choices. Therefore, this methodology is not limited solely to AVs usage intentions but can also be applied to other individual behavioral adoption decisions.

From an individual’s perspective, the influencing factors of usage intention are unveiled. The perception of risk can exert a negative impact on the intention to utilize autonomous vehicles (AVs). This insight can be leveraged to devise more effective promotional strategies for automobile manufacturers, such as showcasing AVs’ driving capabilities and their adeptness in handling hazardous scenarios to instill public confidence in vehicle safety [7], while also leveraging positive social media coverage to highlight the benefits of AVs [54]. Privacy security represents another facet of perceived risk. The deployment of autonomous vehicles should be undertaken within a well-defined context, necessitating proactive consideration of underlying societal issues and values. Consequently, it becomes imperative for governments to formulate corresponding regulations that address concerns like privacy infringement and remote malicious manipulation of others’ vehicles [68].

Additionally, the refinement of regulations is a crucial determinant impacting the intention to utilize AVs. This is consistent with the study of Paschalidis et al. [41], which also demonstrated the impact of regulation refinement on AV usage intention. This has important implications for understanding the social impact of AVS usage intentions. When drivers relinquish control to AVs, it signifies a departure from the existing liability framework [69]. To address liability concerns about autonomous vehicles, it becomes imperative to reformulate the liability structure to ascertain driver accountability. The current legal system places excessive responsibility on drivers, thereby impeding public acceptance and hindering AV development [70].

## 6 Conclusion

In this study, an ordered Probit model with random parameters was employed to analyze the public’s willingness to use automated vehicles, incorporating personality traits, social information, perceived usefulness, perceived ease of use, and perceived risk as key factors. The model was evaluated using survey data from 2105 respondents. The findings revealed that social information, perceived usefulness, perceived ease of use, and perceived risk significantly influenced the intention to use automated vehicles. Notably, perceived usefulness emerged as a stronger predictor of usage intention compared to perceived ease of use. Furthermore, the results indicated that personality variables such as openness and extraversion played a significant mediating role in the relationship between social information, perceived usefulness, perceived ease of use, and perceived risk on usage intention. This study underscores the significance of considering personality traits when examining intentions to adopt new technologies.

Deployment of the Avs will require a concerted effort by automakers and policymakers. Automakers can use media coverage of the safety of self-driving cars to increase people’s willingness to use them. Policymakers can develop better regulations to protect people’s interests and facilitate the deployment of AVs.

There are still some limitations in this study. Studies have neglected other factors that may influence the use of autonomous vehicles. For example, factors such as ethical concerns, privacy concerns, and the environment. Secondly, the survey respondents may not have had experience with AVs, and the survey results depend on their imagination of AVs. Thus, the intention to use AVS is an inclination rather than an actual use. The groups surveyed were not broad enough. Future research can incorporate personal experiences (before and after feelings of using AVs) and automation differences (differences in different AV levels) to further improve the understanding of AV usage intentions.

## Supporting information

S1 Table Results of the confirmatory factor analysis (Internal consistency, reliability and convergent validity of the measurement model) (2022). (DOCX)

S2 Table Results of the confirmatory factor analysis (Internal consistency, reliability and convergent validity of the measurement model) (2023). (DOCX)

S3 Table Estimated results of intention to use automated vehicles. (DOCX)

S4 Table Estimated results of social informativeness (positive). (DOCX)

S5 Table Estimated results of perceived ease of use. (DOCX)

S6 Table Estimation results of perceived usefulness. (DOCX)

S7 Table Estimated results of perceived risk. (DOCX)
