# Supplementary material for: Key factors capturing the willingness to use automated vehicles for travel in China
Source: PLoS One. 2024 Feb 16;19(2):e0298348. doi: 10.1371/journal.pone.0298348 (PMC10871520; doi:10.1371/journal.pone.0298348)
Supplement: S1 Table — (DOCX) [file pone.0298348.s001.docx]

**S1 Table Results of the confirmatory factor analysis (Internal consistency, reliability and convergent validity of the measurement model) (2022).**

| Variable | Items | Convergent validity | | | Cr.α |
| --- | --- | --- | --- | --- | --- |
|  |  | Factor loading | CR | AVE |  |
| The big five | BF1 | 0.679 | 0.908 | 0.496 | 0.904 |
|  | BF2 | 0.684 |  |  |  |
|  | BF3 | 0.73 |  |  |  |
|  | BF4 | 0.681 |  |  |  |
|  | BF5 | 0.722 |  |  |  |
|  | BF6 | 0.711 |  |  |  |
|  | BF7 | 0.705 |  |  |  |
|  | BF8 | 0.706 |  |  |  |
|  | BF9 | 0.727 |  |  |  |
|  | BF10 | 0.695 |  |  |  |
| Social informativeness | SI1 | 0.777 | 0.862 | 0.610 | 0.835 |
|  | SI2 | 0.772 |  |  |  |
|  | SI3 | 0.766 |  |  |  |
|  | SI4 | 0.809 |  |  |  |
| Perceived ease of use | PEU1 | 0.795 | 0.885 | 0.658 | 0.871 |
|  | PEU2 | 0.817 |  |  |  |
|  | PEU3 | 0.803 |  |  |  |
|  | PEU4 | 0.83 |  |  |  |
| Perceived usefulness | PU1 | 0.795 | 0.864 | 0.613 | 0.836 |
|  | PU2 | 0.78 |  |  |  |
|  | PU3 | 0.791 |  |  |  |
|  | PU4 | 0.765 |  |  |  |
| Perceived risk | PR1 | 0.792 | 0.873 | 0.633 | 0.852 |
|  | PR2 | 0.795 |  |  |  |
|  | PR3 | 0.793 |  |  |  |
|  | PR4 | 0.802 |  |  |  |
| Willingness to use | WILL1 | 0.74 | 0.783 | 0.547 | 0.795 |
|  | WILL2 | 0.73 |  |  |  |
|  | WILL3 | 0.748 |  |  |  |
| Note: Cr.α: Cronbach’s Alpha; CR: Composite Reliability; AVE: Average Variance Extracted. | | | | | |
