# Supplementary material for: Key factors capturing the willingness to use automated vehicles for travel in China
Source: PLoS One. 2024 Feb 16;19(2):e0298348. doi: 10.1371/journal.pone.0298348 (PMC10871520; doi:10.1371/journal.pone.0298348)
Supplement: S2 Table — (DOCX) [file pone.0298348.s002.docx]

**S2 Table Results of the confirmatory factor analysis (Internal consistency, reliability and convergent validity of the measurement model) (2023).**

| Variable | Items | Convergent validity | | | Cr.α |
| --- | --- | --- | --- | --- | --- |
|  |  | Factor loading | CR | AVE |  |
| The big five | BF1 | 0.684 | 0.926 | 0.556 | 0.928 |
|  | BF2 | 0.75 |  |  |  |
|  | BF3 | 0.765 |  |  |  |
|  | BF4 | 0.743 |  |  |  |
|  | BF5 | 0.728 |  |  |  |
|  | BF6 | 0.754 |  |  |  |
|  | BF7 | 0.758 |  |  |  |
|  | BF8 | 0.773 |  |  |  |
|  | BF9 | 0.721 |  |  |  |
|  | BF10 | 0.775 |  |  |  |
| Social informativeness | SI1 | 0.779 | 0.859 | 0.603 | 0.857 |
|  | SI2 | 0.776 |  |  |  |
|  | SI3 | 0.752 |  |  |  |
|  | SI4 | 0.799 |  |  |  |
| Perceived ease of use | PEU1 | 0.782 | 0.874 | 0.635 | 0.864 |
|  | PEU2 | 0.798 |  |  |  |
|  | PEU3 | 0.792 |  |  |  |
|  | PEU4 | 0.816 |  |  |  |
| Perceived usefulness | PU1 | 0.77 | 0.864 | 0.613 | 0.86 |
|  | PU2 | 0.779 |  |  |  |
|  | PU3 | 0.782 |  |  |  |
|  | PU4 | 0.801 |  |  |  |
| Perceived risk | PR1 | 0.857 | 0.926 | 0.757 | 0.897 |
|  | PR2 | 0.869 |  |  |  |
|  | PR3 | 0.877 |  |  |  |
|  | PR4 | 0.878 |  |  |  |
| Willingness to use | WILL1 | 0.763 | 0.819 | 0.601 | 0.836 |
|  | WILL2 | 0.786 |  |  |  |
|  | WILL3 | 0.776 |  |  |  |
| Note: Cr.α: Cronbach’s Alpha; CR: Composite Reliability; AVE: Average Variance Extracted. | | | | | |
