# Supplementary material for: Key factors capturing the willingness to use automated vehicles for travel in China
Source: PLoS One. 2024 Feb 16;19(2):e0298348. doi: 10.1371/journal.pone.0298348 (PMC10871520; doi:10.1371/journal.pone.0298348)
Supplement: S3 Table — (DOCX) [file pone.0298348.s003.docx]

**S3 Table Estimated results of intention to use automated vehicles**

|  | constant_1 | constant_2 | constant_3 |
| --- | --- | --- | --- |
| kappa.1 | 0.913*** | 0.971*** | 0.894*** |
|  | (13.230) | (11.032) | (11.492) |
| kappa.2 | 1.832*** | 1.933*** | 1.872*** |
|  | (22.168) | (19.836) | (19.058) |
| kappa.3 | 3.039*** | 2.988*** | 3.048*** |
|  | (30.091) | (28.924) | (24.674) |
| Constant | -0.792* | -1.597*** | -1.016** |
|  | (-1.731) | (-3.485) | (-2.206) |
| Gender | 0.062 | 0.088 | 0.019 |
|  | (0.983) | (1.387) | (0.303) |
| License | -0.381*** | -0.102 | -0.251* |
|  | (-2.786) | (-0.758) | (-1.815) |
| Extroversion | -0.005 | 0.003 | 0.045* |
|  | (-0.181) | (0.131) | (1.681) |
| Agreeableness | -0.015 | -0.004 | -0.041 |
|  | (-0.521) | (-0.133) | (-1.410) |
| Conscientiousness | -0.028 | -0.03 | -0.065** |
|  | (-1.054) | (-1.122) | (-2.386) |
| Neuroticism | -0.015 | 0.05* | 0.005 |
|  | (-0.554) | (1.855) | (0.180) |
| Openness | 0.021 | 0.037 | 0.024 |
|  | (0.756) | (1.330) | (0.835) |
| Aux_experience | -0.201*** | -0.048 | -0.053 |
|  | (-3.012) | (-0.720) | (-0.797) |
| XX | 0.053*** | 0.057*** | 0.067*** |
|  | (5.346) | (5.778) | (6.582) |
| GY | 0.062*** | 0.059*** | 0.056*** |
|  | (7.719) | (6.904) | (6.402) |
| YY | 0.082*** | 0.076*** | 0.087*** |
|  | (8.423) | (7.986) | (8.658) |
| FX | 0.048*** | 0.065*** | 0.048*** |
|  | (4.766) | (6.571) | (4.822) |
| Mean.year | -0.075** | -0.066** | -0.068** |
|  | (-2.520) | (-2.230) | (-2.265) |
| Mean.education | -0.001 | 0.001 | -0.027 |
|  | (-0.039) | (0.019) | (-0.764) |
| Sd.year | 0.01 | 0.009 | 0.005 |
|  | (0.287) | (0.224) | (0.123) |
| Sd.education | 0.055 | 0.002 | 0.061 |
|  | (1.425) | (0.029) | (1.149) |
| Log likelihood | -1632 | -1530 | -1581 |
| *, **, and *** indicate statistical significance at the 10%, 5%, and 1% levels, respectively. | | | |
