# Supplementary material for: Key factors capturing the willingness to use automated vehicles for travel in China
Source: PLoS One. 2024 Feb 16;19(2):e0298348. doi: 10.1371/journal.pone.0298348 (PMC10871520; doi:10.1371/journal.pone.0298348)
Supplement: S4 Table — (DOCX) [file pone.0298348.s004.docx]

**S4 Table Estimated results of social informativeness (positive)**

|  | constant_1 | constant_2 | constant_3 | constant_4 |
| --- | --- | --- | --- | --- |
| kappa.1 | 0.563*** | 0.567*** | 0.603*** | 0.580*** |
|  | (11.032) | (8.413) | (9.644) | (10.171) |
| kappa.2 | 1.365*** | 1.440*** | 1.420*** | 1.449*** |
|  | (21.652) | (17.465) | (18.185) | (19.442) |
| kappa.3 | 2.446*** | 2.434*** | 2.398*** | 2.483*** |
|  | (33.017) | (25.839) | (25.603) | (26.847) |
| Constant | 1.282*** | 1.278*** | 2.094*** | 0.844** |
|  | (3.132) | (2.990) | (4.826) | (2.001) |
| Gender | 0.047 | 0.054 | 0.014 | 0.067 |
|  | (0.763) | (0.835) | (0.209) | (1.048) |
| License | 0.062 | 0.192 | 0.044 | 0.335** |
|  | (0.477) | (1.425) | (0.320) | (2.473) |
| Extroversion | 0.019 | 0.006 | -0.014 | 0.033 |
|  | (0.711) | (0.233) | (-0.505) | (1.229) |
| Agreeableness | 0.025 | 0.023 | -0.023 | -0.001 |
|  | (0.881) | (0.766) | (-0.778) | (-0.051) |
| Conscientiousness | 0.020 | 0.025 | 0.054* | 0.004 |
|  | (0.767) | (0.914) | (1.929) | (0.134) |
| Neuroticism | -0.001 | 0.015 | -0.006 | 0.055** |
|  | (-0.053) | (0.533) | (-0.228) | (2.002) |
| Openness | 0.039 | 0.081*** | 0.032 | 0.082*** |
|  | (1.431) | (2.787) | (1.095) | (2.876) |
| Mean.year | -0.115*** | -0.124*** | -0.144*** | -0.125*** |
|  | (-4.031) | (-4.170) | (-4.771) | (-4.187) |
| Mean.education | 0.003 | 0.009 | -0.015 | -0.023 |
|  | (0.090) | (0.250) | -(0.395) | -(0.638) |
| Sd.year | 0.016 | 0.002 | 0.013 | 0.032 |
|  | (0.393) | (0.030) | (0.368) | (0.707) |
| Sd.education | 0.040 | 0.084*** | 0.108*** | 0.092*** |
|  | (1.412) | (2.922) | (3.892) | (3.236) |
| Log likelihood | -1747 | -1604 | -1679 | -1718 |
| *, **, and *** indicate statistical significance at the 10%, 5%, and 1% levels, respectively. | | | | |
