# Supplementary material for: Key factors capturing the willingness to use automated vehicles for travel in China
Source: PLoS One. 2024 Feb 16;19(2):e0298348. doi: 10.1371/journal.pone.0298348 (PMC10871520; doi:10.1371/journal.pone.0298348)
Supplement: S5 Table — (DOCX) [file pone.0298348.s005.docx]

**S5 Table Estimated results of perceived ease of use**

|  | constant_1 | constant_2 | constant_3 | constant_4 |
| --- | --- | --- | --- | --- |
| kappa.1 | 0.622*** | 0.618*** | 0.665*** | 0.576*** |
|  | (12.508) | (11.271) | (12.359) | (11.398) |
| kappa.2 | 1.290*** | 1.218*** | 1.305*** | 1.261*** |
|  | (18.826) | (17.148) | (20.013) | (18.082) |
| kappa.3 | 2.375*** | 2.255*** | 2.344*** | 2.413*** |
|  | (23.801) | (23.362) | (28.935) | (23.667) |
| Constant | 1.519*** | 1.731*** | 1.766*** | 1.568*** |
|  | (3.556) | (4.067) | (4.281) | (3.606) |
| Gender | -0.125** | -0.023 | -0.071 | -0.060 |
|  | (-1.980) | (-0.363) | (-1.156) | (-0.932) |
| License | 0.098 | 0.159 | 0.098 | 0.042 |
|  | (0.712) | (1.167) | (0.743) | (0.300) |
| Extroversion | -0.039 | 0.025 | -0.021 | -0.020 |
|  | (-1.461) | (0.920) | (-0.817) | (-0.738) |
| Agreeableness | -0.041 | -0.055* | -0.052* | -0.060** |
|  | (-1.415) | (-1.885) | (-1.836) | (-2.012) |
| Conscientiousness | 0.037 | -0.020 | 0.003 | 0.026 |
|  | (1.354) | (-0.734) | (0.131) | (0.914) |
| Neuroticism | 0.033 | 0.024 | 0.036 | 0.018 |
|  | (1.216) | (0.871) | (1.352) | (0.633) |
| Openness | 0.011 | 0.042 | 0.064** | 0.061** |
|  | (0.393) | (1.478) | (2.319) | (2.119) |
| Mean.year | -0.007 | -0.064** | -0.067** | -0.051* |
|  | (-0.248) | (-2.159) | (-2.358) | (-1.686) |
| Mean.education | -0.017 | -0.044 | -0.052 | 0.000 |
|  | (-0.469) | (-1.238) | (-1.497) | (0.012) |
| Sd.year | 0.096** | 0.088** | 0.046 | 0.084** |
|  | (2.405) | (1.973) | (0.960) | (2.122) |
| Sd.education | 0.033 | 0.015 | 0.008 | 0.083** |
|  | (0.653) | (0.336) | (0.161) | (2.053) |
| Log likelihood | -1853 | -1775 | -1788 | -1803 |
| *, **, and *** indicate statistical significance at the 10%, 5%, and 1% levels, respectively. | | | | |
