# Supplementary material for: Key factors capturing the willingness to use automated vehicles for travel in China
Source: PLoS One. 2024 Feb 16;19(2):e0298348. doi: 10.1371/journal.pone.0298348 (PMC10871520; doi:10.1371/journal.pone.0298348)
Supplement: S6 Table — (DOCX) [file pone.0298348.s006.docx]

**S6 Table Estimation results of perceived usefulness**

|  | constant_1 | constant_2 | constant_3 | constant_4 |
| --- | --- | --- | --- | --- |
| kappa.1 | 0.719*** | 0.667*** | 0.603*** | 0.559*** |
|  | (11.872) | (9.468) | (9.492) | (10.722) |
| kappa.2 | 1.539*** | 1.427*** | 1.482*** | 1.433*** |
|  | (18.065) | (15.716) | (17.746) | (20.306) |
| kappa.3 | 2.717*** | 2.538*** | 2.468*** | 2.399*** |
|  | (22.202) | (21.305) | (23.827) | (27.262) |
| Constant | 1.070** | 1.968*** | 2.045*** | 1.471*** |
|  | (2.425) | (4.456) | (4.720) | (3.494) |
| Gender | -0.031 | -0.028 | 0.039 | -0.015 |
|  | (-0.470) | (-0.427) | (0.600) | (-0.239) |
| License | 0.055 | 0.071 | 0.097 | 0.086 |
|  | (0.384) | (0.498) | (0.691) | (0.636) |
| Extroversion | -0.004 | 0.023 | -0.013 | 0.025 |
|  | (-0.146) | (0.821) | (-0.465) | (0.913) |
| Agreeableness | 0.036 | -0.023 | -0.007 | 0.004 |
|  | (1.172) | (-0.770) | (-0.239) | (0.137) |
| Conscientiousness | 0.032 | 0.034 | 0.031 | 0.037 |
|  | (1.121) | (1.187) | (1.115) | (1.361) |
| Neuroticism | 0.002 | 0.000 | -0.016 | -0.012 |
|  | (0.086) | (-0.001) | (-0.587) | (-0.430) |
| Openness | 0.066** | 0.046 | 0.010 | 0.047* |
|  | (2.205) | (1.582) | (0.336) | (1.696) |
| Mean.year | -0.098*** | -0.133*** | -0.084*** | -0.112*** |
|  | (-3.148) | (-4.259) | (-2.753) | (-3.811) |
| Mean.education | 0.041 | -0.039 | -0.014 | -0.056 |
|  | -1.081 | (-1.055) | (-0.389) | (-1.580) |
| Sd.year | 0.076** | 0.089** | 0.100*** | 0.078** |
|  | (2.219) | (2.271) | (2.898) | (2.075) |
| Sd.education | 0.138*** | 0.086** | 0.050 | 0.035 |
|  | (4.298) | (2.517) | (1.261) | (0.932) |
| Log likelihood | -1796 | -1664 | -1710 | -1777 |
| *, **, and *** indicate statistical significance at the 10%, 5%, and 1% levels, respectively. | | | | |
