# Supplementary material for: Key factors capturing the willingness to use automated vehicles for travel in China
Source: PLoS One. 2024 Feb 16;19(2):e0298348. doi: 10.1371/journal.pone.0298348 (PMC10871520; doi:10.1371/journal.pone.0298348)
Supplement: S7 Table — (DOCX) [file pone.0298348.s007.docx]

**S7 Table Estimated results of perceived risk**

|  | constant_1 | constant_2 | constant_3 | constant_4 |
| --- | --- | --- | --- | --- |
| kappa.1 | 0.546*** | 0.492*** | 0.606*** | 0.498*** |
|  | (9.181) | (7.391) | (8.146) | (8.216) |
| kappa.2 | 1.427*** | 1.286*** | 1.418*** | 1.260*** |
|  | (17.458) | (14.279) | (13.313) | (14.099) |
| kappa.3 | 2.522*** | 2.344*** | 2.588*** | 2.298*** |
|  | (23.562) | (20.173) | (17.257) | (18.395) |
| Constant | 1.837*** | 2.364*** | 1.485*** | 2.051*** |
|  | (4.303) | (5.288) | (3.080) | (4.572) |
| Gender | -0.141** | -0.112. | -0.108 | -0.100 |
|  | (-2.183) | (-1.671) | (-1.498) | (-1.518) |
| License | 0.030 | -0.014 | 0.167 | -0.160 |
|  | (0.221) | (-0.100) | (1.078) | (-1.112) |
| Extroversion | -0.013 | 0.021 | 0.054* | -0.003 |
|  | (-0.463) | (0.752) | (1.772) | (-0.118) |
| Agreeableness | -0.009 | -0.053* | -0.014 | -0.004 |
|  | (-0.316) | (-1.744) | (-0.425) | (-0.150) |
| Conscientiousness | 0.005 | 0.037 | 0.054* | 0.043 |
|  | (0.192) | (1.270) | (1.733) | (1.515) |
| Neuroticism | 0.027 | 0.033 | 0.068** | 0.023 |
|  | (0.982) | (1.160) | (2.185) | (0.830) |
| Openness | 0.065** | 0.017 | 0.005 | 0.027 |
|  | (2.251) | (0.554) | (0.157) | (0.910) |
| Mean.year | -0.133*** | -0.199*** | -0.168*** | -0.125*** |
|  | (-4.453) | (-6.285) | (-4.874) | (-4.063) |
| Mean.education | 0.005 | 0.016 | 0.030 | -0.046 |
|  | -0.142 | (0.413) | (0.712) | (-1.215) |
| Sd.year | 0.034 | 0.034 | 0.121*** | 0.076 |
|  | (0.807) | (0.856) | (3.404) | (1.183) |
| Sd.education | 0.097*** | 0.114*** | 0.155*** | 0.080* |
|  | (2.647) | (3.155) | (4.513) | (1.915) |
| Log likelihood | -1686 | -1537 | -1592 | -1659 |
| *, **, and *** indicate statistical significance at the 10%, 5%, and 1% levels, respectively. | | | | |
